# Phenolics over Zeolites and Related Materials—Biomedical and Environmental Applications

**DOI:** 10.3390/antiox13121548

**Published:** 2024-12-17

**Authors:** Bojana Nedić Vasiljević, Marija Takić, Nataša R. Mijailović, Aleksandra Janošević Ležaić, Anka Jevremović, Snežana Uskoković-Marković, Maja Milojević-Rakić, Danica Bajuk-Bogdanović

**Affiliations:** 1 Faculty of Physical Chemistry, University of Belgrade, Studentski Trg 12-16, 11158 Belgrade, Serbia; bojana@ffh.bg.ac.rs (B.N.V.); anka@ffh.bg.ac.rs (A.J.); danabb@ffh.bg.ac.rs (D.B.-B.); 2 Group for Nutrition and Metabolism, Centre of Research Excellence in Nutrition and Metabolism, Institute for Medical Research, National Institute of Republic of Serbia, University of Belgrade, Dr Subotića 4, 11129 Belgrade, Serbia; marija.takic@imi.bg.ac.rs; 3 Department of Pharmacy, Faculty of Medical Sciences, University of Kragujevac, Svetozara Markovića 69, 34000 Kragujevac, Serbia; natasa.mijailovic@fmn.kg.ac.rs; 4 Faculty of Pharmacy, University of Belgrade, Vojvode Stepe 450, 11221 Belgrade, Serbia; aleksandra.janosevic@pharmacy.bg.ac.rs (A.J.L.); snezana.uskokovic@pharmacy.bg.ac.rs (S.U.-M.)

**Keywords:** phenolic compounds, zeolite, antioxidants, porous carriers, pollutants

## Abstract

This work analyzes the following two aspects of zeolite applications: their application as carriers in delivery systems for phenolics applied as antioxidants or anticancer agents and the efficient removal of phenolic compounds from aqueous environments. The dual role of zeolites in increasing antioxidant bioavailability and environmental remediation is summarized, and perspectives on progress in zeolite adaptable applications are given. Special attention is given to theoretical methods that will guide future advanced delivery systems for biomedical applications, as well as serve as a focal point in designing multipurpose materials for comprehensive environmental solutions. Perspectives in both fields are discussed.

## 1. Introduction

The very intense metabolic activity in plants, which includes many compounds, is classified into primary and secondary metabolic pathways. Primary metabolites are responsible for important/basic physiological processes concerning plant growth, development, and reproduction. The secondary metabolic pathway produces compounds that plants need for survival in response to changing environmental conditions. Secondary metabolites represent a response to a stimulating environmental factor, whether positive, such as pollinators, or negative, such as pathogens or stress due to increased UV radiation [1]. Owing to their secondary metabolites, plants have beneficial but also toxic effects on human and animal health [2].

Phenolic compounds, the most abundant secondary metabolites in plants, have at least one hydroxyl group in their chemical structure, and it is directly attached to one or more aromatic rings. They are categorized based on the molecular structure of their aromatic ring into C6 (simple phenols and benzoquinones), C6-C1 (phenolic acids), C6-C2 (acetophenones and phenylacetic acids), C6-C3 (hydroxycinnamic acids, coumarins, chromones, and phenylpropanes), C6-C4 (naphthoquinones), C6-C1-C6 (xanthones), C6-C2-C6 (stilbenes and anthraquinones), C6-C3-C6 (flavonoids, isoflavonoids, and neoflavonoids), (C6-C3)2 (lignans and neolignans), (C6-C3)*n* (lignins), (C6)*n* (phlorotannins and catechol melanins), (C6-C3-C6)*n* (condensed tannins), etc. [3].

Phenolics have significant antioxidant properties, minimizing the harmful effects and oxidative stress imposed by reactive oxygen species and radical compounds [4]. However, phenolic compounds have also been declared to be potentially toxic as they can easily solubilize in aquatic environments, with minimal degradability [5].

By combining phenolic compounds and porous supports, an increase in the availability of active sites for reactive species can be achieved. On the other hand, porous materials can be suitable adsorbents for removing phenolics from water tables [6]. Zeolite and zeolite-based materials present a class of porous materials since they comprise favorable textural properties, thermal stability, and susceptibility to ion exchange processes, and therefore, they represent interesting candidates for phenolics support. Currently emerging are zeolite applications that comprise their antioxidant, antimicrobial, and cytotoxic expressions [7,8,9,10,11,12]. The families of zeolitic materials are numerous and diverse, from natural zeolites to synthesized ones, from low- to high-silica zeolites, from aluminosilicates to aluminophosphates, from microporous to mesoporous materials, and from inorganic and porous to metal-organic frameworks. However, what they all have in common are ordered and uniform porous systems [13,14].

## 2. Phenolics over Zeolites—Biomedical Applications

Recent studies have highlighted the potential of zeolites as carriers for bioactive phenolic compounds, offering solutions to enhance their delivery and stability in biomedical applications. The various aspects of phenolic compounds and the mechanisms through which zeolites can improve their therapeutic efficacy have been explored.

A recently published review by Trendafilova and Popova summarized and discussed findings on how the pore and particle size, structure, and surface modification of silica materials affect the preparation of effective delivery systems for biologically active natural polyphenols. It showed that the structure and appropriate modification of the carrier surface can significantly improve the health benefits of the loaded bioactive molecules [15].

The emerging role of zeolites as support for phenolic compounds may be seen in delivery systems. Recent literature has explored whether the controlled release and stability of a phenolic can be obtained when loaded onto zeolites. The focus relied on the porous structure of the zeolites, which can shield bioactive molecules from degradation and improve their therapeutic efficacy [11].

### 2.1. Polyphenols from Plant Extracts

**Figure 1:**
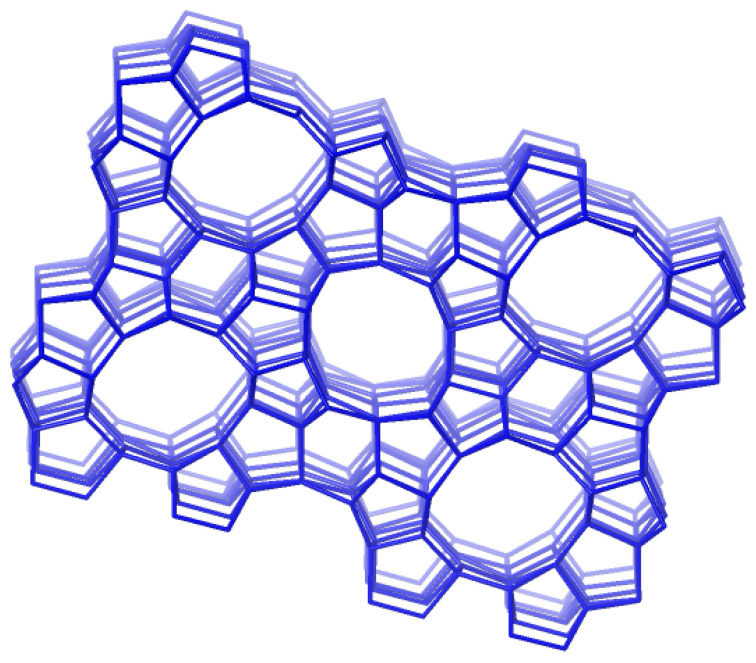
The framework structure of a ZSM-5 zeolite (MFI) [19].

Plant extracts contain large amounts of different polyphenolic compounds, some of the most researched plant chemicals with a wide range of medicinal properties, including antitumor activity. It was shown that the root bark extract of *Hamelia patens* exhibited cytotoxic activity on cervix adenocarcinoma (HeLa) cells [16]. In the crude leaf extract of *Hamelia patens*, chlorogenic acid was present in a high amount (78 µg/g), followed by catechin (26 µg/g), rosmarinic acid (16 µg/g), rutin (14 µg/g), and pyrogallol (10 µg/g). On the other hand, the content of polyphenols in a crude flower extract is much lower, and rosmarinic acid was present in a high amount (5.2 µg/g), followed by p-coumaric acid (3.9 µg/g), chlorogenic acid (2.2 µg/g), p-hydroxybenzoic acid (1.1), and rutin (1.0 µg/g). Encapsulation is an effective technique for protecting these active substances from degradation and losing their activities. The antiproliferative effects of the crude leaf and flower extracts of *Hamelia patens* on different carcinoma cell lines were tested after encapsulation into a ZSM-5 zeolite, serving as a drug delivery system [17]. ZSM-5, or Zeolite Socony Mobil-5, is a member of the Mobil Five framework (MFI), with a ten-membered ring (Figure 1) which forms straight channels of medium pore sizes (∼0.53 × 0.56 nm) and intersecting zigzag channels (∼0.51 × 0.55 nm) [18]. It was shown that the cytotoxic activity of zeolite-based polyphenol delivery systems increased with zeolite loading. Polyphenols were abundant in the extracts, and the antioxidant assay revealed considerable redox activity in these formulations [17].

Polyphenols from plant extracts play an important role in the fight against pathogenic microorganisms. Many studies have shown that these compounds have antimicrobial activity, when using either isolated molecules or plant extracts, and in addition, some polyphenols have shown a synergistic effect when combined with antibiotics and antifungals [20]. The antibacterial activity of silver is well known. The biological effects of silver-zeolite and a polyphenol-containing algae extract were tested in the treatment of oral diseases. The combined impact of silver-zeolite and phenolics for antibacterial and anti-biofilm properties was confirmed. The polyphenol-rich extract alone did not affect bacterial growth but significantly reduced inflammatory cytokine secretion in macrophages. Decreasing lipid peroxidation in gingival epithelial cells revealed the zeolite-supported polyphenol system’s efficiency in treating periodontal diseases [21].

A study in dyslipidemic patients demonstrated that clinoptilolite led to reduced total lipid levels, which was ascribed to its antioxidative action [22]. Clinoptilolite-rich minerals were also tested as carriers for phenolics in ginkgo biloba leaf extract. It was found that flavonoid aglycone constituents selectively adsorbed on clinoptilolite, which led to a decrease in antioxidant activity [23]. This finding supports the idea of phenolic compound delivery from a zeolite carrier, with special attention to a release profile.

### 2.2. Flavonoids and Stilbenoids

**Figure 2:**
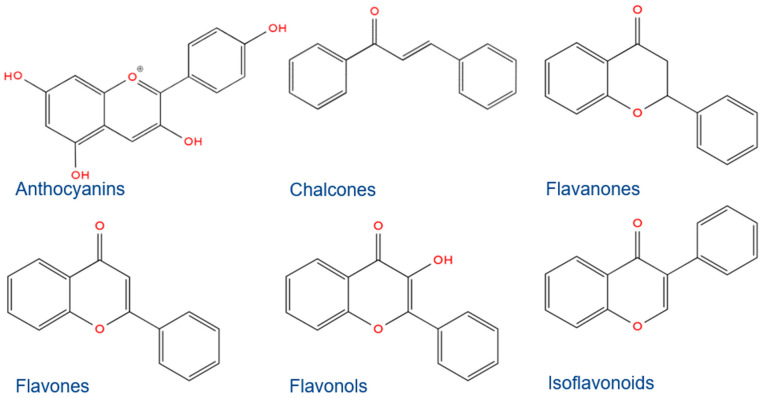
Flavonoid compounds classes.

Flavonoids as polyphenolic compounds are present in various parts of plants (grains, bark, roots, stems, and flowers), fruits, vegetables, teas, and wines, and they have been ascribed positive effects on human and animal health [24]. Their 15C structural framework is organized into two phenyl rings connected by a 3C chain, which usually cyclizes with oxygen to form a third ring. They are classified as anthocyanins, chalcones, flavanones, flavones, flavonols, and isoflavones (Figure 2) [24], each with particular structuring activating selected molecule functionals that they contain, such as their multiple hydroxyl [25] and/or carbonyl groups [26].

**Figure 3:**
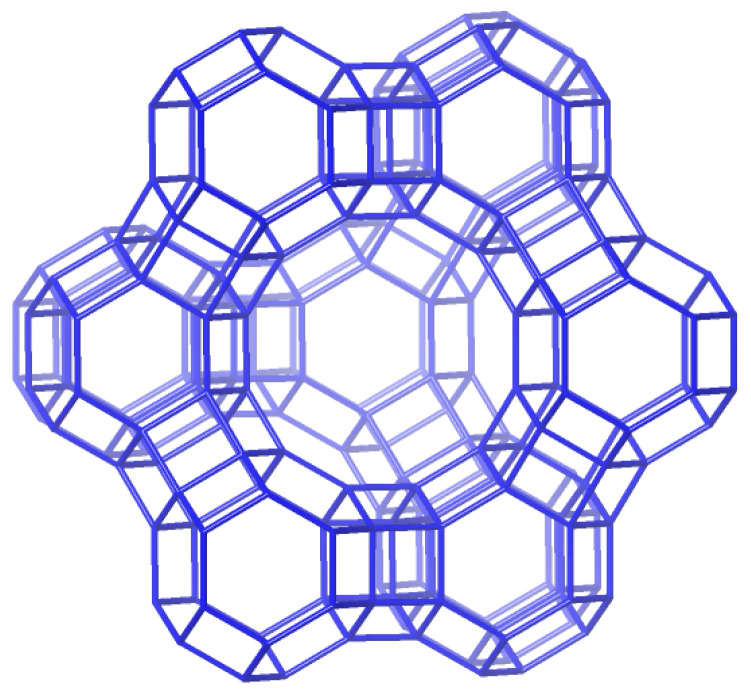
Clinoptilolite (HEU) [19].

Catechins are derivatives of natural polyphenolic compounds known as flavanols, and they are found in abundant concentrations in various fruits and vegetables. Catechin–mineral composite systems offer favorable release kinetics. The microencapsulation and in vitro release of catechin-rich Acacia catechu extract was tested using natural zeolite clinoptilolite (Clinosorbent-5) microparticles [27]. Generally, minerals from the clinoptilolite series (Na, K, or Ca-clinoptilolites) occur in rocks and sediments and are the most common zeolites. These zeolites have a tetrahedral framework labeled HEU, as shown in Figure 3 [19]. In one study, catechin encapsulation reached 32% in an acidic medium with an 88% release efficiency after 24 h in simulated gastric conditions. Since a sustained release profile was achieved, catechin supported on this carrier may be considered for a delivery system without an initial burst effect [27]. 

**Figure 4:**
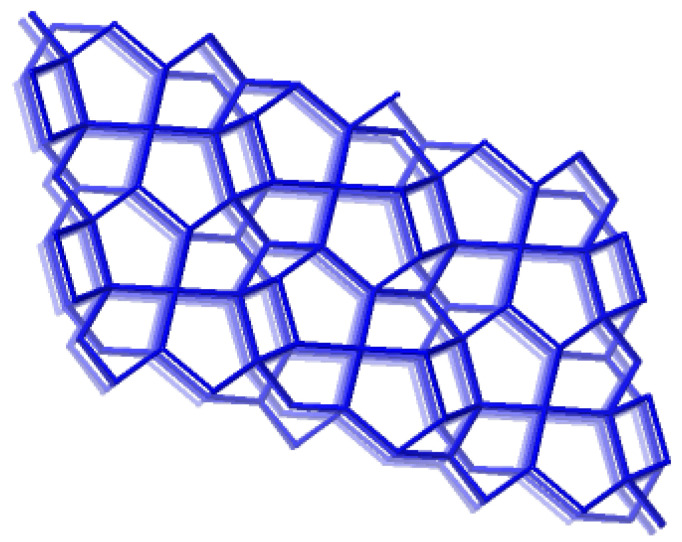
X and Y zeolite (FAU) [19].

Particular interest has been brought to the anticancer properties of epigallocatechin gallate (EGCG), a derivate of catechins (the prevalent polyphenolics of green tea extract). However, effective therapy with EGCG is still limited due to its poor bioavailability and stability in real biomedical applications. To overcome this drawback, different drug delivery systems have been intensively explored to enhance the bioavailability and therapeutic efficacy of EGCG in cancer treatments [28]. Recently, zeolites have also been investigated for this purpose. In addition to natural zeolites, various types of synthetic zeolites have been developed. Zeolite X and zeolite Y are synthetic zeolites belonging to the family of aluminosilicate molecular sieves with a faujasite (FAU) -type structure, as shown in Figure 4. They differ from each other in their Si/Al atomic ratios, which are typically in the range of 1 to 1.5 for X and higher for type Y zeolites [29]. NaX zeolite samples modified with Zn, Mg, Ca, and Sr ions were examined as carriers for EGCG. This polyphenolic forms strong interactions with zeolites but can be sustainably released in a pH-dependent process. A zeolite matrix can establish a shielding effect on EGCG molecules, enabling applications in osteoporosis and cancer treatment. The results of an EGCG release from X zeolites as carriers showed that a system with low doses of EGCG in a neutral pH is promising for osteoporosis medication, while on the other hand, a X zeolite/EGCG system with high doses in an acidic pH may also exhibit effectiveness for cancer treatment [30].

**Figure 5:**
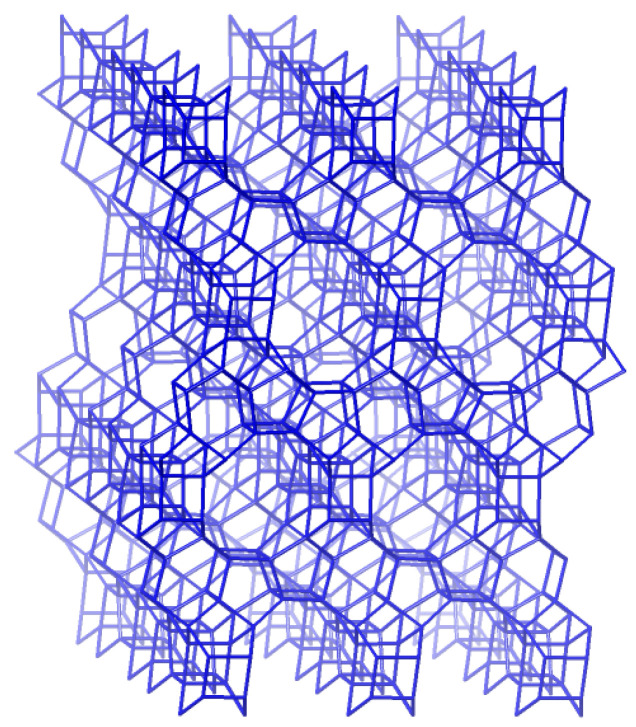
Beta zeolite (BEA) [19].

Resveratrol (*trans*-3,4,5-trihydroxystilbene) is a stilbenoid polyphenol, possessing two phenol rings linked by an ethene bridge, and it can be found in grapes, nuts, fruits, and red wine. This photosensitive compound possesses potential beneficial effects, including antioxidant, anticancer, anti-inflammatory, and cardiovascular-protective properties [31], but it has low chemical stability. The loading of resveratrol in a porous system can stabilize it and protect it from degradation and improve its bioavailability. Among other nanoporous (alumo) silicates (such as MCM-41 and KIL-2), nanosized BEA zeolite was used as a carrier for the preparation of a resveratrol-loaded delivery system. It was shown that its deposition into powdered porous materials stabilized the bioactive *trans*-resveratrol form [32]. BEA zeolite, also known as beta zeolite, consists of a three-dimensional pore system formed by 12-membered ring channels, with diameters of 0.76 × 0.64 and 0.55 × 0.55 nm, and partially disordered structure (Figure 5).

### 2.3. Essential Oils

**Figure 6:**
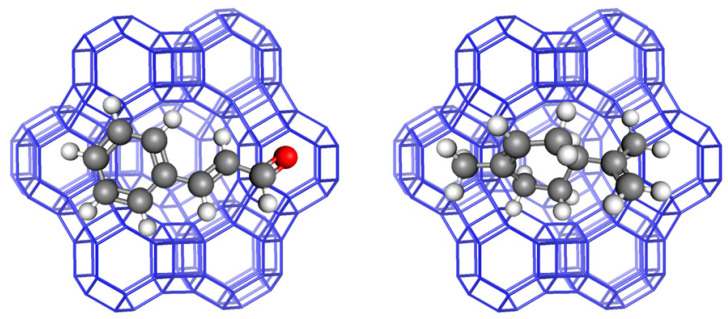
The illustration of encapsulation of cinnamaldehyde and D-limonene in an FAU structure.

Essential oils (EOs), being complex mixtures of volatile compounds composed of terpenoids and phenolic compounds produced by aromatic plants [33], are valued for their natural properties and applications in the pharmaceuticals, cosmetics, and food industries. However, their instability and susceptibility to degradation limit their use. Encapsulating EOs in non-toxic materials like zeolites offers a protective delivery system that can enhance their stability, target delivery, and broaden their applicability. This can provide a cost-effective, non-toxic solution, ensuring sustained EO release and effectiveness [34]. The encapsulation of fragrances present in essential oils in faujasite and mordenite is an example of achieving the desired release dynamic of fragrance components from both carriers with zero-order kinetics; that is, it results in uniform evaporation, independent of the initial loading level. Another study reported the results of release profiles to confirm the protective role of zeolite structures even for a 24-month test period, as shown in Figure 6 [35].

## 3. Phenolics over Zeolites—Environmental Applications

Phenolic compounds are the most common organic pollutants found in wastewater produced by petrochemical industries, coal mining, petroleum oil refineries, and the pharmaceutical, textile, and wood-processing industries. These toxicants are also biologically recalcitrant and persist in wastewater systems at very high concentrations [36].

Simple phenolics induce double-strand DNA breaks, DNA adducts, mutations, and chromosome aberrations, and some of them induce precancerous lesions, papillomas, and cancers, acting as cocarcinogens and exerting a promoting effect in various rodent assays [37]. Phenol is highly irritating to the skin, eyes, and mucous membranes and is toxic if ingested. In animal studies, oral exposure to phenols has caused reduced fetal body weights, growth delays, and developmental abnormalities in offspring. In humans, anorexia, weight loss, diarrhea, dizziness, and blood and liver issues may occur with prolonged exposure. The EPA has classified phenol as a member of Group D as it is not classifiable regarding its potential to cause cancer in humans [38].

The use of zeolites as adsorbents stems from their microporosity and uniform pore size. From the point of view of the adsorption process, an ideal adsorbent has a large adsorption capacity and is selective, easily regenerated, durable, stable in relevant conditions, affordable, and modifiable for achieving optimal properties. Zeolitic adsorbents possess very promising properties and potential applicability [39]. There is active research focused on new zeolite structures and modifications/functionalization for solving the current problems of remediating water systems via adsorption [40]. Additionally, zeolite composites with conducting polymers can serve as efficient phenol-sensing electrodes [41].

### 3.1. Adsorption of Simple Phenols

A study by Uçar et al. evaluated the use of natural zeolite clinoptilolite and its acid-activated form for adsorbing phenol, o-, p-, and m-chlorophenol. The maximum adsorption capacities were below 10 mg/g, with a higher affinity for chlorophenols than phenol itself [42]. The ion exchange procedure introduces novel active sites in a zeolite framework. Cobalt-modified clinoptilolite has proven highly effective in phenol removal, especially under acidic conditions. The formation of a phenol–cobalt complex has been proposed as it binds to a negatively charged zeolite surface, resulting in the removal of 50% 100 mg/L of phenol concentrations [43]. In another study, an additional form of surface modification relied on surfactants, where the adsorption capacity of over 80 mg/g for phenols was witnessed in the case of carbonized clinoptilolite-rich tuff modified with octadecylammonium ions [44].

Fly ash, the principal industrial waste byproduct from the burning of solid fuels constituted mainly of unburned carbon and metal oxides (Si, Fe, Ca, and Al), can be used as a precursor in the production of zeolite [45]. Zeolite synthesized from fly ash and its chitosan-modified form were shown to be effective for the simultaneous removal of Cu(II) and phenol, which are often found in wastewater [46].

Carbon materials, such as activated carbon, are the most widely used of all adsorbents due to their high specific surface area, adsorption capacity, microporous structure, and surface reactivity. Therefore, the adsorption capacities of other materials are often compared with theirs. Levi et al. compared the efficiency of LTA zeolite for phenol adsorption with the adsorption performance of carbonaceous materials [47]. The main building blocks of LTA zeolite (Linde Type A), also known as zeolite A, are sodalite cages linked by four-membered rings, forming a three-dimensional network [48]. The possibility of efficient water decontamination was witnessed for LTA–clay composites in the removal of over 89% of the chlorophenol and 95% of the nitrophenol that were present [49].

### 3.2. Adsorption of Phenolic Acids

**Figure 7:**
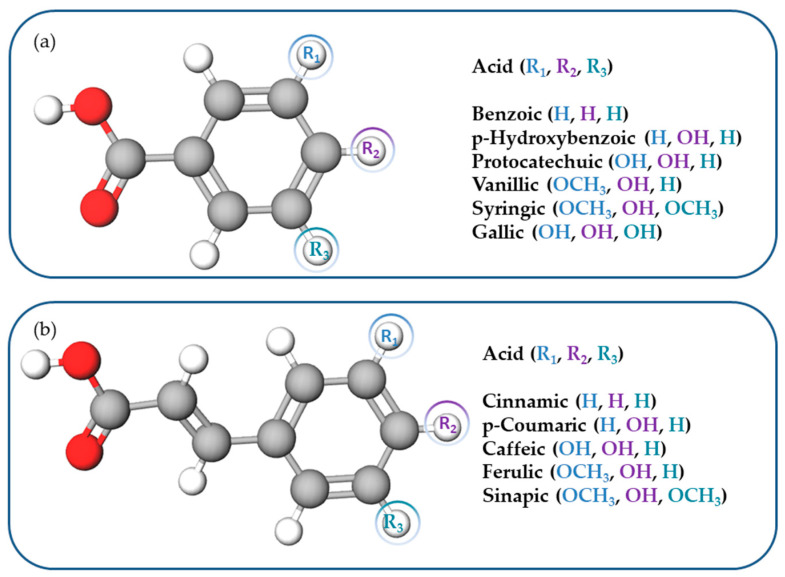
Structure of some polyphenolic acids derived from (**a**) benzoic and (**b**) cinnamic acids.

Phenolic acids (PAs) are phenols or polyphenol derivatives containing at least one hydroxyl group attached to an aromatic ring, and they occur in the form of amides, esters, and glycosides. PAs comprise derivatives of benzoic and cinnamic acids, i.e., hydroxybenzoic and hydroxycinnamic acids, respectively [50]. Figure 7 summarizes the most important representatives of the two classes.

**Figure 8:**
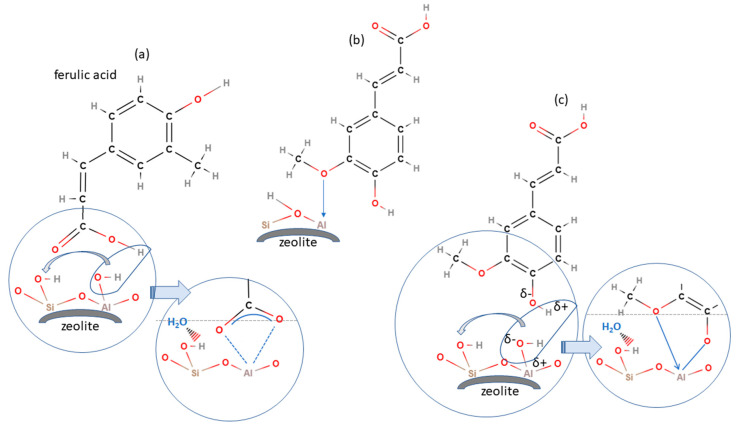
Illustration of the ferulic acid interaction with zeolite via (**a**) COOH-groups, (**b**) CH_3_O-groups and (**c**) via aromatic functional groups.

Hydroxycinnamic acids are cinnamic, caffeic, p-coumaric, cafeic, ferulic, and sinapic acids, while hydroxybenzoic acids are benzoic, p-hydroxybenzoic, protocatechuic, vanillic, gallic, and syringic acids. Due to the radical scavenging activity of numerous hydroxyl groups, PAs are known as potent antioxidants [51], which places them in a number of biomedical applications [50]. The protection against oxidative stress exerted by these compounds enables their application in studies on treating cancer and cardiovascular and neurodegenerative conditions. Additionally, PAs exhibit anti-inflammatory effects by modulating key signaling pathways and inhibiting the production of pro-inflammatory mediators. Their antimicrobial activity has been examined in infection treatment and wound healing. Numerous scientific findings on their therapeutic benefits have led to different pharmaceutical formulations and supplementation with PAs. The adsorption of ferulic acid on H-form FAU, MFI, and BEA zeolite was studied as a step in the thermal transformation of a lignin model compound, where the HY (FAU) zeolite achieved a 90% adsorption in 10 g/L under acidic conditions. Pazo-Cepeda et al. reported that ferulic acid interacted through its phenol and carboxyl groups [52]., forming monodentate and bidentate surface complexes, as illustrated in Figure 8. Our group has extensive experience with the oxygen and nitrogen-rich compounds that are used to target biomedical and environmental areas. We have employed spectroscopic and semi-empirical calculations to confirm that hydrogen bonding with surface hydroxyls on a zeolite framework is the dominant interaction mechanism [8,9,10,11]. Therefore, the anticipated binding mechanism for different phenolic compounds predominantly involves physisorption, a process which mainly takes place through dipole–dipole intermolecular interactions. Additionally, the establishment of chemical or coordination bonds could hinder the sustained release mechanism, which is critical in biomedical applications and would make the regeneration process significantly more energy-intensive in the environmental field.

An FAU composite membrane was prepared using a ceramic substrate derived from coal fly ash to separate vanillic acid and phenols. The maximum separation efficiencies of 79% for vanillic acid and 89% for phenol indicated that the zeolite membrane was highly effective for separating different phenolic compounds [53].

Linh et al. studied the adsorption kinetics of phenolic compounds on BEA zeolite [54]. The selection was made according to the different critical diameters of the non-volatile phenolics, such as p-coumaric acid, ferulic acid, and vanillin [54]. These compounds exhibited reduced mobility on zeolite surfaces compared to the volatile compounds. The importance of the kinetic separation of phenolic compounds in zeolites based on their differing diffusivities was essential for their removal and recovery.

### 3.3. The Role of the Si/Al Ratio and Charge-Balancing Ions on Zeolite Performance

In the case of synthetic zeolites, the expansion of the Si/Al ratio and the control of Al distribution are highly desirable because, in this way, better activity, selectivity, and durability of the zeolite can be achieved [55]. Phenol adsorption from wastewater on zeolitic adsorbents may be enhanced by high Si/Al ratios in a zeolite framework [56]. In this study, FAU zeolite showed better performance in the competitive binding of water and phenol than BEA or MOR zeolites, i.e., mordenite, another important class of large-pore zeolites (Figure 7). In turn, a high Si/Al ratio BEA and MFI were more efficient adsorbents than activated carbon. Khalid et al. suggested that the hydrophobicity of the zeolite was key to its adsorption properties. For instance, siliceous BEA zeolite was found to be an effective and regenerable adsorbent for phenol removal at concentrations below 1.6 g/L, maintaining its properties even after ten cycles [56].

Not all applications are sensitive to the Si/Al ratio. Beta zeolite (BEA) adsorbents (with Si/Al 35 and 150) were compared to commercial polyvinylpolypyrrolidone and polymeric amberlites for polyphenols recovery [57]. Both beta zeolite samples showed similar efficiencies and reached a capacity of 203 mg/g for ferulic acid. This was the highest adsorbed value among the tested caffeic, sinapic, p-coumaric acid, and catechins. Experiments with rapeseed meal and wheat seed extracts showed that a 7.5% zeolite suspension could recover 100% of the major polyphenols, and regeneration with isopropyl alcohol under mild conditions did not induce a loss in adsorption/desorption capacities, which enabled BEA to be further tested in polyphenols isolation from renewable sources [57].

Charge-balancing ions play an important role in zeolite performance. NaY zeolites and their Ni-modified form were tested for phenol removal from water. The introduction of Ni by an ion-exchange procedure increased adsorption to 89% compared to the starting sample. The hydrogen bonding was proposed as the primary interaction mechanism, as is often witnessed for adsorbates with phenol functionalities. Reusability testing showed that the performance stability was maintained across five cycles for the FAU samples [58].

### 3.4. Surface and Porosity Modification of Zeolite

To enhance the interaction between zeolites and phenolic adsorbates, a number of modifications to zeolite surfaces may be employed, along with the preparation of composite materials. Surfactant-modified zeolite samples derived from coal fly ash were tested for removing ionizable and non-ionizable phenolic compounds [59]. A surfactant bilayer increased adsorption significantly, especially for phenol, p-chlorophenol, and bisphenol A at higher pH levels, while aniline, nitrobenzene, and naphthalene were adsorbed consistently across the pH range. Adsorption efficiency correlates with the hydrophobicity of the molecules, as hydrophobic contaminants are more readily retained by a modified zeolite. Cationic surfactants were also employed for the modification of an FAU zeolite, which improved the adsorption of tannic acid through π–π and hydrophobic interactions [6]. FAU-type zeolites modified with cationic surfactants (cetylpyridinium chloride, tetrapropylammonium chloride, and benzalkonium chloride) also effectively co-adsorbed the insecticide acetamiprid, while the zeolite and tannic acid reduced insecticide toxicity, opening a new application for zeolite/phenolics systems in environmental remediation [6]. Following the potential of zeolites for co-adsorption, Khalil et al. explored the selective removal of phenols from a biofuel mixture of alkanes and aromatic compounds [60].

Phenols differently adsorb in micropores and mesopores. Recently, adsorbents were selected among zeolites, silica-based porous materials, alumina, and activated carbon, and their capacities were tested in view of their textural properties. The phenols were adsorbed in micropores by condensation in supercages, while in mesopores, the interactions relied on silanol groups. Strong acidic sites in faujasites enhanced phenol adsorption over toluene but limited regeneration. Optimal results were achieved with slightly dealuminated zeolites, which developed both micro- and meso-porosity [60].

Advances in material science have enabled the production of nanosized zeolitic materials. Decreases in the sizes of zeolite crystals leads to considerable increases in their external surfaces and consequently affects their surface charge, hydrophilicity/hydrophobicity, and surface ion-exchange. In this way, there are new possibilities for exploring the adsorption and reaction of large molecules that do not normally interact with microporous zeolites [61]. Fe-nano zeolites were proposed as an adsorbent for removing phenolics from wastewater with higher capacities compared to activated carbon [62]. The efficiencies achieved were 139 mg/g for phenol, 159 mg/g for 2-chlorophenol, and 171 mg/g for 2-nitrophenol [62].

A very interesting application of phenol adsorption on zeolites is in the recovery and selective separation of phenolics from industrial waste. Waste from wine production is rich in phenols and other organic compounds, and it has a harmful effect on both flora and fauna. Selective recovery from wine industry wastes preserves phenolic contents with unaffected antioxidant activity. Treatment of these extracts with zeolites and aluminum oxide, pre-treated with NaOH and CH_3_OH, respectively, were shown to separate up to 93% of the phenolics from the total sugars [63].

## 4. Theoretical Basis of Interaction Between Phenolics and Carriers

The adsorption processes of phenolic acids on different adsorbents have been poorly studied using density functional theory (DFT) methods, including molecular dynamics methods. Advances in view of interactions between phenolics and their supports may be the route for obtaining specifically designed porous carriers. Unfortunately, the interactions between phenols and zeolites have not been considered in the existing literature. The study by Hernández-Tamargo et al. can be highlighted, in which molecular dynamics (MD) simulations and density functional theory (DFT) calculations were performed as a complement to quasielastic neutron scattering experiments in studying the mobility of phenols in beta zeolites. The translational activation energies between 26 and 35 kJ mol^−1^, calculated from the MD simulations, indicated that the translational diffusion was too slow to be observed and determined experimentally. The rotational activation energies in the acidic and all-silica zeolites implied that the molecules not bound to the acidic sites were responsible for the rotational dynamics in the MD simulations, indicating that a portion of the phenols was hydrogen bonded to the acidic sites, while the remainder underwent isotropic rotation in the H-beta catalyst pore [64].

However, since theoretical tools are essential in this quest for optimal loading, whether biomedical or environmental applications are envisaged, we considered calculations for porous supports that have been investigated in the literature, although they were not intrinsically related to zeolites. 

So far, studies that have included these advanced tools mainly dealt with the adsorption properties of benzoic acid (BA). The adsorption of BA on various surfaces, particularly single-walled carbon nanotubes (SWCNTs), has been studied using DFT calculations that have provided insights into the electronic properties and stability of BA when adsorbed on these nanostructures. For instance, Li et al. [65] demonstrated that the adsorption capacity of BA on SWCNT bundles could reach as high as 375 mg/g, significantly surpassing traditional adsorbents like activated carbon. This high capacity was attributed to the presence of high binding energy sites within the SWCNT structure, which enhanced the adsorption process. In this study, molecular dynamics simulations were conducted to examine the adsorption behavior of a 25% benzoic acid solution around carbon nanotubes (CNTs). The system was equilibrated using an NPT ensemble (an isothermal-isobaric ensemble as follows: number of particles, pressure, and temperature are all constant) at 298 K, and carbon nanotubes were inserted into a cylindrical void within the solution. Two types of single-walled nanotubes (SWNTs) with different chiralities were simulated for sixteen nanoseconds. The adsorption behavior, influenced by factors like curvature and groove size, was analyzed using Visual Molecular Dynamics (VMD) software [66] and the Large-scale Atomic/Molecular Massively Parallel Simulator (LAMMPS), with bonded and non-bonded interactions modeled via the all-atom optimized potentials for liquid simulations (OPLS-AA) force field and Extended Single Point Charge (SPC/E) water model. The results showed that the BA was adsorbed on almost the entire surface of the aromatic ring on the nanotube, indicating flat adsorption.

Arsano et al. [66] simulated very similar systems of benzoic acid and carbon nanotubes and observed that the amount of BA retained decreased with increased surface modification due to the loss of aromatic adsorption sites. This suggested that optimal surface characteristics were key to maximizing the adsorption efficiency. The highly functionalized carbon nanotubes retained less benzoic acid due to the loss of aromatic adsorption sites. However, the adsorbing molecules showed stronger binding, suggesting that the carboxylation aided adsorption through hydrogen bonding, complementing the dominant aromatic interactions.

An example of a theoretical study of the adsorption of benzoic acid on another substrate is the work of Hacklell et al. [67], which investigated the adsorption of benzoic acid on the (110) surface of rutile (TiO_2_), comparing unreconstructed and (1 × 2) reconstructed surfaces using density functional theory (DFT). The study examined the effects of hydrogen bonding and van der Waals interactions on the stability of different adsorbate configurations. Through local density approximation (LDA), generalized gradient approximation (GGA), and DFT-D2 methods, the research highlighted the differences between the computational and experimental results, especially regarding the orientation of the benzene ring in the adsorption geometry.

Van der Waals forces significantly affect the adsorption of carboxylic acids on TiO_2_ surfaces, with a more significant binding effect seen in acids with aromatic side chains. This study demonstrated the importance of using an exchange-correlation functional in DFT calculations considering vdW interactions.

Recent research by Zhang et al. [68] focused on investigating the antioxidant and photophysical properties after gallic acid adsorption onto graphene quantum dots. DFT, time-dependent DFT, and MD were performed for this investigation. The simulated absorption spectra showed that the gallic acid/graphene quantum dot complex effectively covered both the UVA and UVB regions with reduced fluorescence intensity, making it potentially superior for sun protection compared to GA alone. In addition, the dynamic simulations revealed that both the gallic acid and the complexes spontaneously reacted with the hydroxyl radicals, highlighting their potent antioxidant properties. The modelling results for the gallic acid adsorption on the graphene quantum dots showed that weak van der Waals forces were established. The gallic acid molecule was positioned almost parallel to the quantum dot, which suggested the presence of a π-π interaction.

## 5. Perspectives

The scarcity of works dealing with phenolics immobilization has opened up a novel investigation spotlight for porous carriers. Encapsulated or supported polyphenols have been shown to improve stability and bioavailability compared to free compounds, making them promising for both in vitro and in vivo applications [69]. While many existing heterogenization technologies have been applied to polyphenols in the biomedical field or removal by adsorption for environmental applications, this field remains underexplored. The fundamental results in molecular interactions may overcome the current challenges, including varying evidence for disease treatment and high costs for industrial-scale production. Investigations such as DFT and related theoretical calculations may offer insights into the intrinsic energy of interactions, molecular orientations, and favorable active sites for phenolics retention. Furthermore, adsorption and loading capacity, adsorption/desorption kinetics, and thermodynamics are necessary for acquiring basic information about these systems. It is expected that the samples active in cytotoxicity studies may be potent in antioxidant activity and, in turn, adsorption performance. The potential for zeolite-based materials to be regenerated and reused is crucial for sustainable environmental applications and will be an integral part of these studies. The materials studies addressing the different potencies of the selected heterogeneous phenolics will unquestionably comprise theoretical investigations alongside experimental research. Future studies will likely focus on hybrid materials, functionalized porous carriers, improved delivery systems, and new stabilization strategies, enhancing the role of zeolites and related materials in functional pharmaceuticals and environmental solutions.

## 6. Conclusions

Here, we summarize the multipotent applications of zeolites, focusing on their potential to address issues related to human health and environmental pollution through innovative material design. The analysis of zeolites’ roles in advanced carriers for bioactive molecules, such as antioxidants and anticancer agents, is compared to their efficiency in the removal of phenolic compounds from aqueous environments. This dual functionality is rarely seen in material science, and the value of zeolites is recognized in improving the bioavailability and stability of phenolic compounds, thus enhancing their therapeutic potential.

Despite there being some research on zeolite-based systems, the field of phenolic immobilization remains relatively underexplored, offering a promising route for further investigation. The encapsulation or support of polyphenols within porous carriers like zeolites has revealed improvements in stability and bioavailability compared to their free forms. However, this field has its challenges, including high costs and inconsistencies in evidence for therapeutic applications, limiting the broader application of these technologies. The mechanisms of adsorption of phenolic substances on zeolites and related materials have been examined from the viewpoint of how different types of zeolites/porous carriers and their modifications impact efficacy. Additional issues with more efficient zeolite regeneration methods and the development of hybrid materials that combine zeolites with other functional materials also need to be addressed.

The integration of theoretical approaches, such as density functional theory (DFT) and related calculations, is anticipated to play an important role in future studies by providing insights into molecular interactions, associated binding energies, and the identification of optimal active sites for phenolic retention.

Future research is expected to focus on enhancing adsorption capacities, describing adsorption/desorption kinetics, and exploring process thermodynamics for developing more efficient systems. The synergy between theoretical insights and experimental findings will be critical in developing novel zeolite-based systems with improved functionality. The expected advancements will not only enhance the role of zeolites in pharmaceutical applications but also contribute to comprehensive environmental research, making zeolites a versatile tool in both health and environmental solutions.
